# Innovative Extraction Technologies for Development of Functional Ingredients Based on Polyphenols from Olive Leaves

**DOI:** 10.3390/foods11010103

**Published:** 2021-12-31

**Authors:** Maria Lisa Clodoveo, Pasquale Crupi, Alessandro Annunziato, Filomena Corbo

**Affiliations:** 1 Interdisciplinary Department of Medicine, University of Bari “Aldo Moro”, P.zza Giulio Cesare 11, 70124 Bari, Italy; marialisa.clodoveo@uniba.it; 2 Department of Soil, Plant and Food Sciences, University of Bari “Aldo Moro”, Via Orabona 4, 70125 Bari, Italy; alessandro.annunziato@outlook.it; 3 Department of Pharmacy-Drug Science, University of Bari “Aldo Moro”, Via Orabona 4, 70125 Bari, Italy; filomena.corbo@uniba.it

**Keywords:** biophenols, industrial waste, green technologies, secoiridoids, energy saving

## Abstract

Olive tree (*Olea europea* L.) leaves represent around 10% of the total weight of olives arriving at any given mill, which are generally discarded, causing economic and environmental issues. However, these are rich sources of natural bioactive compounds (i.e., polyphenols), which have health-promoting potential. Thus, the valorization of olive leaves by recovering and reusing their components should be a must for food sustainability and circular economy. This review provides an insight into the principal polyphenols present in olive leaves, together with agronomic variables influencing their content. It also summarizes the recent advances in the application of novel extraction technologies that have shown promising extraction efficacy, reducing the volume of extraction solvent and saving time and cost. Moreover, potential industrial uses and international patents filed in the pharmaceutic, food, and cosmetic sectors are discussed.

## 1. Introduction

Approximately 8 million ha are cultivated with olive trees (*Olea europaea* L.) in the Mediterranean countries, accounting for almost 98% of the world crop in 2017 [1]. Olive leaves represent one of the main by-products derived from both olive tree cultivation and the olive-processing industry. Leaves are accumulated in large volumes on farms during the pruning of the trees; generally, they represent 10% of the weight (around 300–750 kg/ha or 25 kg per olive tree) of olives collected for oil extraction, although the quantities may vary depending on culture conditions, tree age, production and/or local pruning practice [2,3,4]. Moreover, olive mill leaves are separated during the cleaning of olives by a blower machine; around 76 and 133 kg/ha/year have been generated in 2017 considering the data from FAOSTAT (2019) [5]. 

This huge and cheap amount of residues has no practical applications, and indeed is usually burned, ground, and disposed of in landfills or occasionally removed for animal feed [6]. It is then directly thrown away as a by-product, potentially causing economic and environmental problems and wasting a resource, with increasing cost for producers due to its removal, storage, and elimination [7]. Nevertheless, the olive leaf could be used to obtain value-added products, including antioxidants, oligosaccharides, protein, lignin, and biofuels, through the implementation of chemical and biochemical processes [5,6]. This could provide a promising option to increase the profitability of olive groves and make agricultural activity practices more sustainable [8]. 

Over the years, there have been many proposals for the evaluation and exploitation of agricultural and industrial leftovers [9], which in many cases can yield higher contents of bioactive compounds than the final product does [10]. Olive leaves are quite relevant in this context because they are a potentially inexpensive, renewable, and abundant source of phenolic substances (such as oleuropein, verbascoside, rutin, tyrosol, and hydroxytyrosol), very similar to those present in olives and their derived products [11,12,13,14,15,16], which display biologic activities including antioxidant, antimicrobial, and antiproliferative properties [17,18,19,20,21]. The extensive quantitative and qualitative phenolics pattern of olive leaves varies according to many factors, including olive variety, climate conditions, age and biological cycle of the olive tree, as well as agricultural practices [11,22,23,24]. Olive leaves have long been known for their therapeutic and medicinal properties. It is claimed that they might aid in the treatment of a broad range of infectious diseases caused by bacteria and viruses [25,26,27]; indeed many studies, both in vitro and in vivo, have proved their important biological effects, including being radio-protective, anti-fungal, anti-atherosclerotic, hypoglycemic, cardioprotective, and cytotoxic on cancer cells [28].

Therefore, there is certainly a growing interest in the valorization of such by-products for the recovery and/or biotransformation of their organic matter and the use of olive leaf powders or extracts in various industrial applications such as food supplements, cosmetics, and the pharmaceutical industry [29]. For this reason, more cost-effective and sustainable extraction methods to prepare olive leaf extracts have been designed in the last few years, rather than using purification methods such as organic solvent fraction and column chromatography [23]. Different extraction techniques have been used to isolate phytochemicals in olive leaves, ranging from the more traditional (such as dispersed-solid liquid extraction, percolation, and Soxhlet) to non-conventional emerging technologies (such as microwave and ultrasound-assisted extraction and supercritical fluid extraction) in order to favor mass transfer, shorten extraction times, and/or reduce solvent requirements [23,30]. Moreover, the technological parameters (i.e., particle size, solvent type and composition, solid-to-solvent ratio, extraction temperature and pressure, extraction time, and pH) affecting olive leaves’ biophenolic profile and extractability have been carefully optimized [31]. 

This review focuses on olive leaves’ functional compounds (especially polyphenols) and the extraction methods for their recovery, as well as the potential industrial uses in the pharmaceutical and food sectors of olive leaves and extracts.

## 2. Olive Leaves Chemical Composition

Several factors (e.g., sampling period, cultivar, age of olive tree, and climatic changes) can influence olive leaf composition [24]. The organic matter in olive leaves is variable (76.4–92.7 g/100 g dry matter) and accounts for around 38% of their weight [32]. The crude protein and polypeptides content is low (2.49–10.9 g/100 g dry matter), while the amino acid quantity (89.9 g/100 g total nitrogen) is relatively important [23,33]. The carbohydrate composition includes glucose, fructose, myo-inositol, galactose, galactinol, sucrose, raffinose, stachyose, and starch fractions [34]; in particular, mannitol, a very important polyol, is present in significant concentrations (10 and 20 g/kg olive), which means that its extraction from olive leaves can be considered as an interesting alternative to its commercial production by chemical hydrogenation of sugars [29]. Terpenes and lipophilic compounds are also present (2.28–9.57 g/100 g dry matter) [23]. The main triterpene from the olive leaf is oleanolic acid (30 g/kg dried matter), followed by significant concentrations of maslinic acid and minor levels of α-amyrin, ursolic acid, erythrodiol, and uvaol [35]. The literature on the biological activity of oleanolic acid is extensive, and its pharmacological importance has been highlighted [36]. All of these considerations amply justify the recovery of oleanolic acid from the olive leaf. The other lipophilic compounds include saturated hydrocarbons, squalene, wax, tocopherols, triglycerides, β-carotene, chlorophyll, linear alcohols, and fatty acids (palmitic, oleic, and linoleic acids) at ppm level [37]. In addition, olive leaves contain ample amounts of potassium, manganese, magnesium, and copper [31]. Furthermore, there are essential oils with antioxidant activity, consisting of a very complex mixture of aldehydes, ketones, esters, alcohols, alkenes, and alkanes, whose major constituents are 2-E-decenal (20.43%), benzeneacetaldehyde (4.00%), 2-undecenal (3.71%) and valencen (3.31%) [38].

**Figure 1:**
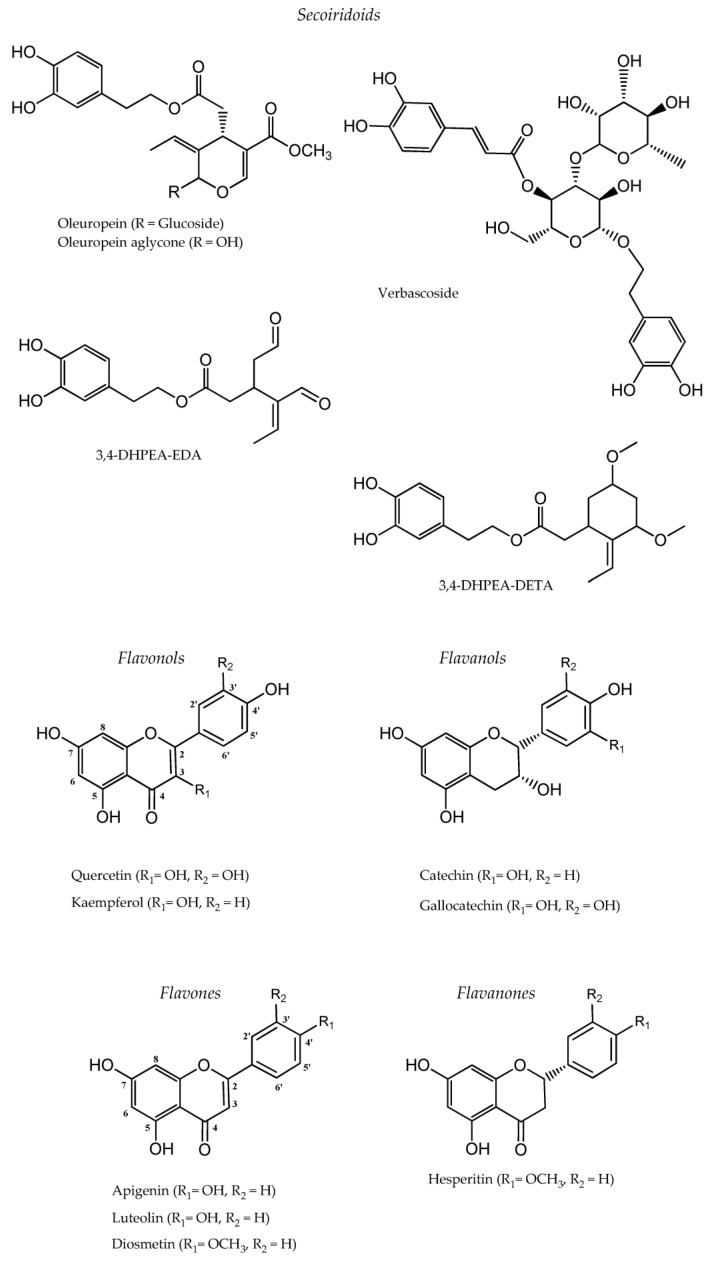

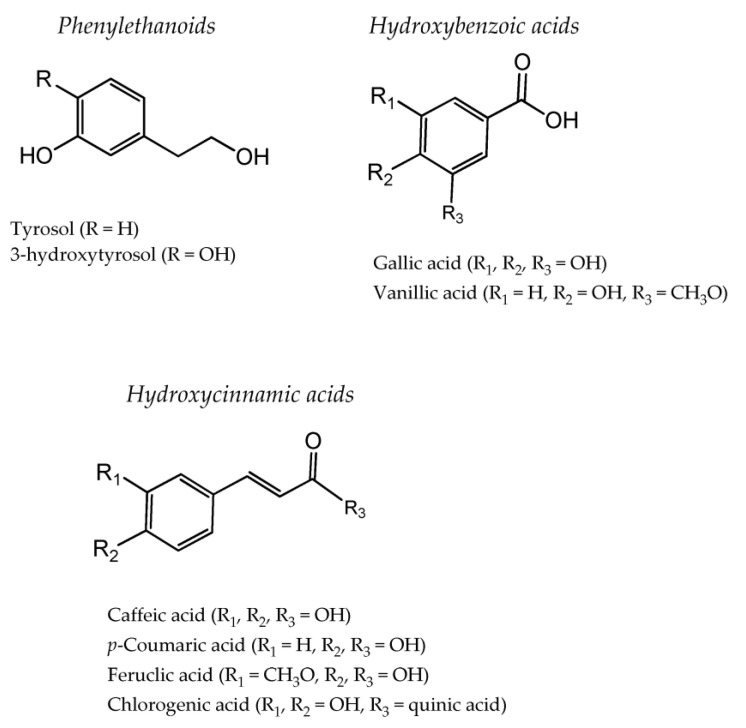
Typical structures of phenolic compounds identified in olive leaves.

However, despite the significant amount of the aforementioned nutrients present in olive leaves, major attention is devoted to their phenolic fraction (e.g., secoiridoids, flavonoids, and simple phenols), which ranges from 15 to 70 mg/g fresh weight, because of its health benefits and bioactivity [24]. In addition to their diversity (Figure 1), phenolic compounds are found in olive leaves at different concentration levels. The ranges of individual phenolic compounds contents in the literature are reviewed in Table 1. 

### 2.1. Secoiridoids, Tyrosol, and Hydroxytyrosol Derivatives in Olive Leaves

As amply recognized, secoiridoids are specifically restricted to the *Oleaceae* family and represent quantitatively the flagship compounds in olive leaves [39,40]. The prominent constituent is oleuropein (around 8–14% dry leaf weight), responsible for the bitter taste of the fruits and leaves of olive plants, which together with the products of its enzymatic or chemical hydrolysis have the greatest biological interest [32,41]. The protective attributes of oleuropein are reflected typically by their inhibiting effects against oxidation, microbial disorders, inflammation, and platelet aggregation [42]. Oleuropein aglycone, deriving from oleuropein de-glycosylation in olive leaves, has a great contribution to developing bitterness/astringency and health benefits associated with its ability to curtail neurodegeneration, decrease low-density lipoprotein cholesterol, and promote the oxidative stability of lipids [42]. Lower contents of verbascoside, 2-(3,4-hydroxyphenyl) ethyl (3S, 4E)-4-formyl-3-(2-oxoethyl)hex-4-enoate (3,4-DHPEA-EDA), and oleuroside are also present in olive leaves depending on the cultivar [43]. Recently, a new secoiridoid (3,4-DHPEA-DETA) was isolated from an olive leaf [32].

### 2.2. Flavonoids and Simple Phenols in Olive Leaves

Olive leaves have been found to be a robust source of flavonoids. Total flavonoids ranged from 56 to 125 mg/g catechin, equivalent in olive leaves of nine Tunisian cultivars, and from 21 to 55 mg/g on a dry basis depending on the extraction techniques [44,45]. They can be present in the aglycone form (quercetin, apigenin, luteolin, diosmetin, kaempferol, and hesperitin) or in the glycosylated form (quercetin-7*-O-*rutinoside, luteolin-7*-O-*rutinoside, luteolin-7*-O-*glucoside, luteolin-4′*-O-*glucoside, luteolin-7,4′*-O-*diglucoside, apigenin-7*-O-*glucoside, apigenin-7*-O-*rutinoside, and quercetin-3*-O-*rutinoside) [24,46]. Apigenin-7*-O-*glucoside and luteolin-7*-O-*glucoside were the major flavonoids identified in Chemlali olive leaf cultivars, while eight flavonoids have been identified and quantified in 18 Portuguese olive leaf cultivars by a reversed-phase HPLC-DAD procedure. Flavonoids exert a favorable protection against carcinogenic, cardiovascular, and microbial diseases [42].

Simple phenols (e.g., hydroxytyrosol, tyrosol, caffeic acid, vanillic acid, gallic acid, chlorogenic acid, *p*-coumaric acid, and ferulic acid) are present in olive leaves in lower amounts than secoiridoids and flavonoids [46]. The hydroxytyrosol content of olive leaves, using the high-performance liquid chromatography (HPLC) method, has been reported to be around 2.28 mg per 100 g leaf extract, while tyrosol is usually present in a trace amount (0.0007 mg/g dry leaves) [47]. Hydroxytyrosol and the other simple phenols generally offer dietary health benefits in terms of antioxidation, anti-atherosclerosis, anti-carcinogenic, and anti-inflammation [42].

## 3. Varietal and Agronomic Variables Influencing Functional Compounds Content

Olive cultivar, geographical origin, maturity stage, climate, tree/leaf lifetime, as well as sampling/harvest time and storage are typical pre-harvest and post-harvest agronomical and technological factors affecting the phenolic compounds and overall chemical composition of olive leaves [42,66]. In a 2016 study, olive leaves total polyphenols were found to vary from 7.87 to 34.21 mg/g depending on the kind of leaf (fresh, refrigerated, dried, frozen, or lyophilized), cultivar, sampling time, and production area [74]. Leaves from 20 olive cultivars (San Felice, Galleaga, Frantoio, Maurino, Luccino, Arbasana, Arbequina, Empeltre, Koroneiki, Taggiasca, Grappolo, Manzanilla, Ouslati, Santa Caterina, Ligurian, Itrana, Pendolino, Sevillano, Chitoni, Picual, Chemlali, Coratina, Mission, Aglandau and Nichitskaia) collected from San Antonio, Texas, in successive seasons were characterized by variable levels of oleuropein, verbascoside, luteolin-7*-O-*glucoside, and luteolin-4′*-O-*glucoside [75]. Then, in general, the oleuropein concentration was significantly higher in the cold season and in the tropical regions than in the hot season and the temperate regions [76].

Geographical origin strongly influenced the total phenolic content (from 7.35 to 38.66 mg-GAE/g-dried leaf) present in olive leaves from Anatolian cultivars dislocated in six different sites; there appeared to be a general trend for polyphenols to decrease in the samples of the trees cultivated in windy and humid air [77]. Moreover, biophenol content has effectively been used to classify and discriminate cultivars of olive trees (Alameño, Arbequina, Azulillo, Chorna, Hojiblanca, Lechín, Manzanillo, Negrillo, Nevadillo, Ocal, Pierra, Sevillano, and Tempranillo) from the same cultivation zones, and of Arbequina olive trees cultivated in different geographical zones (Córdoba, Mallorca, Ciudad Real, Lleida, and Navarra) [78].

A study on antioxidant capacity change through leaf maturation showed that mature leaves have more antioxidant activity than young leaves [25]. Bouaziz and Sayadi [79] found low variations of oleuropein concentration in olive leaf extract during the harvest period (from 12.4 to 14.2%), and its content decreased with increasing leaf maturity [39]. Olive cultivars may have a different response from biotic or abiotic factors, consequently leading to distinct olive leaves’ phenolic profiles and antioxidative activity [24]. Oleuropein concentration and antioxidant activity in Drobnica, Leccino, Levantinka, and Oblica leaves increased following exposure to low air temperatures, which may be of crucial interest for designing more efficient and sustainable phytochemical farming strategies [66]. Leaves from olive trees continuously exposed to environmental stresses such as high temperature and UV radiation have produced phenolic alcohols, secoiridoids, and flavonoids, whose quantitative and qualitative changes depended on cultivar, maturation degree of the leaf, infestation caused by Dacus olea, climate, and geographical origin [32].

Additionally, leaf nutrient status, recorded for various olive cultivars grown in the same environment at different sampling times, have demonstrated a great impact on olive leaves’ phenolics, since nutrient availability is essential for providing co-factors for many enzymes of their biosynthesis pathways [80,81]. Combined foliar application of Mg, Mn, and B resulted in a significant decrease of flavonoid content in olive leaves [82], conversely B nutrient deficiency stress resulted in higher levels of specific secoiridoids [83].

## 4. Extraction Technologies of Functional Compounds from Olive Leaves

Extraction from fruit and vegetable matrices is generally the critical step for both the isolation and exploitation of their bioactive compounds; therefore, finding suitable extraction techniques is a noteworthy phase in the valorization of agriculture leftovers and transformation residues like olive leaves. Ideally, an extraction method should be quantitative, non-destructive, and time-saving; moreover, it should be selected on the basis of the desired ingredients, extract composition and purity one wishes to obtain [42,84]. On the other hand, using the extract without isolating the constituents might be recommended to achieve health benefits because of the synergistic effects of all biophenols present in the extract [31].

For instance, the extraction system may effectively enable the exertion of de-glycosylation and/or the hydrolysis of oleuropein to hydroxytyrosol, which is appreciably prized for its biological activity and functionality [23]. However, if oleuropein is the desired final product, the system should be optimally designed to ensure recovery of the target pure/intact molecules with minimum degradation/chemical reaction. In this regard, the control of parameters affecting the extraction efficiency (such as preliminary preparations, particle size of the extracted material, solvent type, solvent composition, solid to solvent ratio, extraction temperature and pressure, extraction time, and pH) is mandatory given the high susceptibility of the compounds, as well as for commercial applications of the process [42,85]. The oleuropein yield from olive leaves was equal among 3 min of microwave-assisted extraction, 2 h of supercritical CO_2_ fluid extraction, and 6 h of Soxhlet extraction [7].

Conventional extraction by maceration in organic solvents has been traditionally used to extract phenolic compounds from olive leaves. Hydroalcoholic solvents such as methanol or non-toxic ethanol/water mixtures have been shown to be the best choice for extracting both lipophilic and hydrophilic phenols [86,87]. Nonetheless, the main disadvantages of this process include low extraction efficiency and the length of time needed in solid–solvent contact to reach equilibrium; using high temperatures does lead to a kinetic improvement, but this is limited by the fact that polyphenols are sensitive to high temperatures. As a consequence, in order to solve these limitations, new techniques have been developed in recent years for the extraction of bioactive compounds from plant materials, including ultrasound-assisted extraction, microwave-assisted extraction, pressurized liquid extraction, and supercritical fluid extraction [88]. In term of selectivity, microwave-assisted extraction and conventional solvent extraction in general seem to be more suitable for extracting polar compounds such as oleuropein derivatives, apigenin-rutinoside, and luteolin-glucoside isomer. Meanwhile, supercritical fluid extraction or pressurized liquid extraction are more efficient in extracting compounds with less polarity such as apigenin, luteolin or diosmetin [23].

### 4.1. Drying Methods

Since olive leaves’ bioactive compounds are very sensitive to environmental stresses, post-harvest treatments such as drying operations for biomass stabilization may be crucial to allow the maximum recovery of biophenols [89]. The selection of a suitable drying approach is important to render the moisture unavailable for microbial/enzymatic activity and oxidation reaction [90]. In this sense, immediate drying before the extraction of olive leaves is the primary operation in maximizing their exploitation by limiting their perishability and susceptibility to spoilage and thus increasing the amount of phenolic compounds and the antioxidant capacity of the extracts [32,49]. Drying temperature and time are the main parameters which influence drying performance and consequently the concentration of the target polyphenols in the extracts [42]. Air-drying in the shade could be an appropriate, simple and low-cost procedure for olive leaf preservation without any detrimental effect on the nutritive values. Drying of leaves at room temperature (25 °C) fully preserves oleuropein and verbascoside levels, while drying at an elevated temperature of 60 °C results in loss of different polyphenols to various levels [32]. Moreover, a study conducted exploring a specific drying temperature from 65 to 80 °C revealed a declining concentration of oleuropein in the leaves, thus proving that high temperatures in prolonged drying operations may generally destroy or alter the structure of bioactive ingredients [91]. Nonetheless, notably, Kamran et al. [92] recommended olive leaves should be dehydrated at 105 °C for 3 h before extraction to obtain the maximum recovery of oleuropein and other biophenols. However, hot air drying (120 °C for 8 min) provided a higher phenolic content than freeze-drying, especially in oleuropein. Freezing reduced the antioxidant potential as compared to fresh leaves, probably due to oxidase activation, although its influence was not dependent on the freezing method [49,60].

Different types of dryers have been used over the years for the purpose of dehydrating olive leaves, including the pilot scale heat pump conveyer dryer, tray dryer, thin layer dryer, convective laboratory solar dryer, and freeze dryer [32,93,94,95,96]. Microwave, infrared, and vacuum methods have also been applied to dry olive leaves for the extraction of bioactive compounds, with the advantages of reducing drying time and energy requirement and preserving a fresh green color [32,63,97].

### 4.2. Traditional Extraction Technologies

Percolation, solid-liquid extraction (e.g., maceration, Soxhlet extraction), distillation, and heat reflux are examples of conventional extraction techniques, commonly employed for recovering biophenols from olive leaves [42]. They involve a large proportion of organic solvents, the choice of which strongly influences the extraction yield; moreover, they use agitation and/or high temperature to maximize the diffusivity/mass transfer of the desired compounds from the matrix [85]. It should be noticed that both these thermal and non-thermal procedures are also time consuming, having high energy or production cost, and possibly giving lower-quality extracts because of prolonged extraction time and the use of high temperatures [42]. There are many reports on the investigation of the parameters affecting the traditional processes, both qualitative and quantitative. With regards to the solvent type, ethanol, methanol, ethyl acetate, boiling water, hexane, diethyl ether, chloroform, and butanol were the main solvents used. In particular, methanol mixture (i.e., 80%) is recommended for extracts with high levels of flavonoids and polyphenols from olive leaves [98]; however, this may lead to unacceptable levels of toxic residues in the final extracts, especially if they are targeted for human use [51]. Ethanol and water are alternative green solvents for this purpose. The boiling of dried leaves in deionized water adjusted to pH 3 at 60 °C for 4 h has shown the highest extraction recoveries for oleuropein and verbascoside from olive leaves [99]. Conversely, pure ethanol was not effective as a solvent for the extraction of phenolics, whilst ethanol/water mixtures increase diffusion and solubility of the compounds by altering the density, dynamic viscosity, and dielectric constant of the solvent [85]. 80% aqueous ethanol (*v/v*) has been reported as the optimum solvent for the extraction of secoridoids and flavonoids from olive leaves [23,30].

A range of extraction temperatures and times were examined in conventional extractions; generally, less extraction time is required with increasing temperature, which causes a decrease of solvent viscosity and favors the diffusion rate of the compounds and extraction yield [12,30]; however, high temperatures may also degrade the phenolics [85]. A solvent to solid ratio of between 10 and 50 was mostly reported in the literature [23,100]. Finally, as for the pH effect, increasing the pH from 2 to 6 provoked an almost linear decline in biophenol extraction [31].

### 4.3. Non-Coventional Extraction Technologies

Over recent years, many attempts have been made to develop modified/advanced technologies for the efficient extraction of bioactive compounds from olive leaves [42]. In the literature, a range of emerging technologies using less or no organic solvents have been proposed to date as alternative tools for the intensification of the recovery of polyphenols while maintaining their chemical integrity and, subsequently, their functional activities [88] (Table 2).

#### 4.3.1. Ultrasound Assisted Extraction (UAE)

UAE is considered one of the most interesting techniques, being an efficient and inexpensive alternative to conventional extraction procedures. Ultrasound enhances the extraction rate by increasing the mass transfer and possible rupture of cell walls due to the well-known “cavitation effect”, leading to higher product yields without modifying the extract composition. UAE reduces processing time, thermal degradation losses, solvent and energy consumption; moreover, and is compatible with any solvent [88,109]. Indeed, for instance, UAE could also be applied in the enhancement of extraction with water as well as with other generally recognized as safe (GRAS) solvents [110].

Several researches have shown how UAE is able to improve the extraction yield of phenolic compounds from olive leaves, such as oleuropein, verbascoside, luteolin-4′*-O-*glucoside, luteolin*-7-O-*glucoside, apigenin-7*-O-*glucoside, and quercetin-3*-O-*rutinoside, by markedly shortening the extraction time without provoking significant changes in the structural/molecular properties and functionality of most biophenols [43,62,69,73]. UAE coupled with reduced pressure has also been demonstrated to further improve the extraction of oleuropein from Frantoio olive leaves in terms of yield and extraction time [7].

The influences of extraction time, temperature, solvent concentration, solid to liquid ratio, particle size, ultrasound power, and frequency were investigated in several studies. Extraction temperatures ranging from 30 to 50 °C together with extraction time around 50–60 min have been employed as optimal operating conditions [62,73,101,102]. Wang et al. [73] achieved the maximum yield of flavonoids at 270 W ultrasound power and 41 mL/g liquid-solid ratio, while a high oleuropein yield was obtained operating at 600 W and 30 mL/g [43]. Furthermore, an improved yield of oleuropein, verbascoside, and luteolin-4′-*O-*glucoside (32.6%, 41.8%, and 47.5%, respectively) compared to conventional solid extraction was found at a liquid-solid ratio of 15 mL/g [62]. Regarding solvent type and concentration, different preferences appear in the literature, from 50% acetone to 50–70% ethanol, for the best recovery of oleuropein, phenolic acids, phenolic alcohols, and flavonoids from olive leaves [31,58]. However, natural deep eutectic solvents (i.e., choline chloride-fructose-water) have proved to efficiently extract oleuropein, caffeic acid, and luteolin [111].

#### 4.3.2. Microwave Assisted Extraction (MAE)

MAE is one of the most advanced methods currently used in extracting polyphenols from olive leaves. Depending on the polarity of the solvent and presence of ions in the solvent, both mechanisms of dielectric heating and ionic conduction can occur simultaneously; solvents with high or medium absorbance capacity of MW, such as methanol, ethanol, water, or their mixtures must be used. The advantages of MAE are mainly quick heating, extraction efficiency, lower solvent requirements, short extraction time, and a clean process. On the other hand, the scale-up of MAE systems may represent major drawbacks, as the length of penetration of microwaves is rather limited [42].

Extraction time, temperature, type of solvent, solvent concentration, sample to solvent ratio, and microwave power are the main factors influencing the recovery of polyphenols using MAE. However, MAE is usually performed at higher temperatures (>80 °C), and therefore its application in the isolation of antioxidants has to be carefully assessed. The MAE of Tunisian olive leaves under optimal conditions of methanol-water (80:20, *v*/*v*), at a temperature of 80 °C for 6 min, have revealed the highest recovery of methoxyoleuropein, diosmin, luteolin-diglucoside, and luteolin-rutinoside [86]. Further, MAE has also been used in the extraction of oleuropein from dried olive leaves after microwave irradiation at 800 W for 10 min [23]. MAE, at 86 °C for 3 min with water as the solvent, was found to be the most efficient approach, giving rise to an increase of 82% in the recovery of total phenolics (over the conventional maceration method), especially when incorporated as a pre-treatment step during the UAE of olive leaves [103].

Optimum MAE conditions (extraction time, liquid/solid ratio, and MW power) were recently examined using response surface methodology (RSM) with Box–Behnken design (BBD) or Central Composite Design (CCD) for the better extraction output of total phenolic content from olive leaves, taking water as a green solvent [112,113]. Besides, new highly efficient and truly eco-friendly processes combining sustainable deep eutectic solvents (i.e., choline chloride derivatives) were proposed for recovering phenolic compounds from leaves and general olive oil processing wastes [50,61]. An irradiation power of 250 W, an extraction time of 2 min, and an amount of sample 5 g were the extraction conditions, optimized through RSM and artificial neural networks (ANN) for the maximum recover of total phenolics (~2.48 μg/mL) and oleuropein (~0.06 μg/mL) from olive leaves after solvent-free MAE [104].

#### 4.3.3. Supercritical Fluid Extraction (SFE)

The basic principle of SFE is that at the critical point (a specific temperature and pressure) a fluid behaves like a single phase, retaining the properties of gas and liquid simultaneously. At this condition, fluid diffuses into the solid matrix like a gas and dissolves active materials like a liquid. The most attractive fluid for SFE is CO_2_ because it is non-toxic, non-flammable, chemically inert, cheap, and available in high quality and in high quantities [42]. Moreover, CO_2_ has a very low critical temperature (31 °C), and therefore it can be easily removed, allowing rapid and selective extraction [114]. SFE using CO_2_ is widely exploited for the green extraction of polyphenols from olive leaves, even though the process needs the inclusion of a polar solvent (modifier) to help increase the solubility in CO_2_ and the extraction yield [42]. Indeed, Taamalli et al. [86] found that SFE-CO_2_ extraction of phenolics from the leaves of six Tunisian olive varieties was more efficient in ghd extraction of compounds with less polarity such as apigenin, luteolin, or diosmetin compared to MAE and UAE. Meanwhile, regarding the obtaining of the best oleuropein yield (14.26 mg/g dried leaf), Aegean dried and ground olive leaves were extracted by using CO_2_ modified by methanol at 300 bar and 100 °C in the SFE method [105]. Another study has showed that SFE at 300 bar with 60% ethanol as a co-solvent proved to be the best option for obtaining a high-activity antioxidant extract from olive leaves [106]. Plaza et al. [107] have recently proposed two hydrophilic deep eutectic solvents, CIS-DES (a 1:1 mixture of choline chloride and citric acid) and Etagline (a 1:2 mixture of choline chloride and ethylene glycol), combined with supercritical CO_2_ extraction, for recovering hydroxytyrosol from olive mill waste. The only serious drawback of SFE is the system complexity and the higher investment cost as compared to traditional atmospheric pressure extraction techniques.

#### 4.3.4. Pressurized Liquid Extraction (PLE)

PLE works on the principle that boiling point temperature is proportional to pressure. Therefore, the pressure of the extraction system is increased before raising the temperature (usually in the range of 50–200 °C), in order to keep the solution in a liquid state. Several researchers have proven that the solubility of polyphenols in solvents is increased in PLE; higher amounts of polyphenols are recovered from solid matrices (such as olive leaves) at elevated temperatures, even though the maximum temperature of extraction is dependent on both the solvent and compound thermolability [88]. Notably, the process is energy saving and uses ecofriendly and non-toxic solvents (mainly water and aqueous alcohols), and its extraction equipment is very simple [88].

The number and duration of extraction cycles, pressure, solvent volume to sample mass ratio, and especially temperature and solvent type are the variables affecting the extraction efficiency. PLE has been applied to olive leaves in order to obtain the optimal oleuropein extraction by a mixture of H_2_O/EtOH (43:57) at 190 °C for 1 extraction cycle [23]. Analogously, PLE using an ethanol-water (60:40, *v/v*) mixture at 190 °C for 5 min has been shown to be a viable technique for extracting oleuropein, luteolin-7*-O-*glucoside, apigenin-7*-O-*glucoside, quercetin, and verbascoside (up to 120, 4.65, 3.01, 0.75 and 2.23 g/kg dry weight, respectively) from olive leaves with a lower consumption of solvents and lower energy costs [53]. In a recent study, extraction with PLE was conducted isobarically (10.3 MPa), varying the temperature and solvent; the highest total extract yield (30.91%) was obtained at 60 °C using ethanol and water (80:20, *v:v*), whilst the highest concentration of total flavonoids, oleuropein, and antioxidant activity (82.87%) was obtained at 60 °C using just ethanol [108].

#### 4.3.5. Pulsed Electric Field extraction (PEF)

PEF assisted extraction consists of the application of short-duration pulses (μs to ms) of mild electric voltage (namely 0.5–20 kV/cm) to a sample placed between two electrodes. This treatment can determine an irreversible electroporation, which leads to the mechanical destruction of the cell membrane, favoring the extraction of secondary metabolites [115]. PEF has been extensively investigated as a non-thermal food processing, food preservation, and microbial inactivation technique. On the other hand, the use of PEF in the recovery of bioactive compounds from by-products is less explored, although it is attracting a growing interest as a field of study.

Recent studies have been published on the optimization of PEF as a standalone “green” technology for the extraction of high value-added compounds from fresh olive leaves; these showed how optimal detected PEF contributes to increasing the value (~30%) of the total polyphenols assayed by Folin-Ciocalteu method and, particularly, in increasing the recovery (~120%) of luteolin-diglucoside, quercetin-3*-O-*rutinoside, luteolin-rutinoside, luteolin-7*-O-*glucoside, apigenin-7*-O-*rutinoside, luteolin-3′*-O-*glucoside, luteolin aglycone, and oleuropein determined by HPLC, transpired using a rectangular-shaped extraction chamber and a 25% *v/v* aqueous ethanol solvent choice, using a pulse duration of 2 μs under 0.85 kV/cm electric field strength, and a period of 100 μs for a 15 min extraction duration [64,65].

#### 4.3.6. Other Techniques

Superheated liquid extraction (SHLE) technique is based on using aqueous or organic solvents at high temperature and pressure without reaching the critical point. It can be applied in static mode (with a fixed volume of extractant), dynamic mode (where the extractant flows continuously through the sample), and static-dynamic mode (a combination of the above two modes). The low costs of acquisition and maintenance as well as the use of low-toxic solvents make the industrial implementation of this extraction method very advisable. In static-dynamic SHLE approaches, high amounts of oleuropein, hydroxytyrosol, and tyrosol (up to 23,000, 2800, and 1500 mg/kg, respectively), as well as other biophenols such as verbacoside, apigenin-7*-O-*glucoside, α-taxifolin, and luteolin-7*-O-*glucoside, have been extracted from olive leaves [23].

Infrared-assisted extraction is a new eco-friendly technology that enhances the extraction of bioactive compounds from natural matrices using a ceramic infrared emitter; it is easy to use, economical, requires low energy consumption, and has great potential to be scaled-up to an industrial level. Under optimal conditions, the total phenolic content (in particular oleuropein and hydroxytyrosol) yielded from olive leaves is enhanced by more than 30% using IR, as contrasted with water bath traditional extraction, which even requires 27% more ethanol consumption [67].

High voltage electrical discharge (HVED) is an emerging green electro-technology which can be suitable for the efficient extraction of biophenols from olive leaves. The advantages of HVED include its non-thermal nature, selective extraction, shorter extraction time (<10 min), and clean process (water or ethanol as solvent) [51].

## 5. Possible Industrial Uses

Olive leaf extracts, rich in polyphenols, have found various industrial applications, involving the food, cosmetic, or pharmaceutical sectors. Some examples are examined in the following sections, as well as relevant patents (2011–2021), which are listed in Table 3.

### 5.1. Food Industry

In the last few years, mainly due to the health benefit and nutraceutical activity related to the phenolic composition, olive leaf extracts have been patented by the food industry as foodstuffs or food additives for producing functional foods with health properties (Table 3). The inclusion of phenolic-rich olive leaf extracts in the food system, as an alternative functional source compared to the expensive purified biomolecules such as oleuropein, offers the advantage of being a low-cost means of processing as it eliminates the need for the purification step, while being considerably effective.

To date, olive leaf extracts are commercially available as dried leaves, powders, extracts, or tablets used as herbal teas or food supplements [24]. Extracts of hot freshwater leaves are eaten to increase diuresis and treat hypertension and bronchial asthma [28]. Furthermore, olive leaf-derived phenolic compounds have shown significant antimicrobial properties, thus playing an important role in the control of food processing and preservation during storage, as well as in counteracting pathological microorganisms such as Helicobacter pylori and other food-borne pathogens [116]. The natural antioxidants from olive leaves are cost effective and efficient preservatives that can extend the shelf life of food products and prevent losses of their sensory and nutritional qualities in the food industry. For instance, the enrichment of refined olive and refined olive-pomace oils with oleuropein-, oleuropein aglycone-, and hydroxytyrosol-rich extracts has been proven to inhibit the deterioration of oil rancidity by improving stability and shelf-life [24]. An ultrasound approach for the direct enrichment of edible oils (olive, sunflower, and soya) with the main biophenols in olive leaves was developed [31]. Recently, spray-drying micro- and nano-encapsulated olive leaf extract was recommended for controlling the oxidative stability of processed food [117,118]. Olive leaf extracts have been used in different technological and functional applications, such as biodegradable films for food packaging [119], meat and meat products, and fruit and fruit derivatives [120], or in biscuit formulations as a strategy for the mitigation of dietary advanced glycation end products [121]. Because of the astringent and bitter taste of polyphenols, several studies and patents have formulated olive leaf extract-fortified food products by reducing their bitterness (Table 3) [32]. Olive leaf extracts also have potential applications in dairy products, due to their ability to increase the nutritional value of fermented milk (i.e., yogurt) without affecting the viability of lactic acid bacteria [70].

### 5.2. Pharmaceutic and Cosmetic Industry

In the last decade, several studies and patents have been developed on medical supplements containing olive leaf as a liquid extract or in tablet form against diabetes, high blood pressure, cardiovascular and neurodegenerative diseases, the common cold, urinary tract infections, and chronic fatigue syndrome, as well as to support the immune system (Table 3) [59,122,123,124,125]. Products containing olive leaf extract have also been used for their anti-ageing activities in the cosmetic industry (Table 3). It is worth pointing out that, to date, the most effective olive leaf products on the pharmaceutical market derive from leaf extracts containing the natural biophenols that work together in natural synergy to maximize the health benefits [31,72]. García-Villalba et al. [20] illustrated how the intake of whole olive phenolics extracts in postmenopausal women could prevent age-related and oxidative stress-related processes such as osteoporosis. Oleuropein and hydroxytyrosol from olive leaf extracts have proved to be a unique class of HIV-1 inhibitors, effective against viral fusion and integration; moreover, olive leaf polyphenols have inhibited vitro platelet activation in healthy and non-smoking males [23].

Oleuropein-, oleuropein aglycone-, and hydroxytyrosol-rich extracts have shown hypocholesterolemic effects; indeed, they have reduced serum total cholesterol, triglycerides, and low density lipoprotein-cholesterol levels as well as slowing down the lipid peroxidation process [126]. Thanks to the synergistic effect of oleuropein, hydroxytyrosol, and flavones such as diosmetin or luteolin, a crude extract of olive leaves has been found to inhibit the cell proliferation of human breast adenocarcinoma, human urinary bladder carcinoma, and leukemic cells [23]. Oral administration of olive leaf extract and oleuropein twice a day for 14 days has contributed to reducing the risk of skin thickness induced by ultraviolet B radiation [127].

Interestingly, a recent study has reported the specific biological effects of three new functional infusions based on different mixtures of the leaves of three olive cultivars (Istarska bjelica, Buža and Leccino) designed to protect against cardiovascular diseases, reduce the risk of diabetes mellitus, and exert antimicrobial activity [128]. The use of olive leaf extracts as a reducing agent in the synthesis of gold and silver nanoparticles is becoming widely applied in the pharmacological sector, especially for controlled drug delivery and as a safer alternative to conventional antibacterial agents [129,130].

## 6. Sustainability and Circular Economy

**Figure 2:**
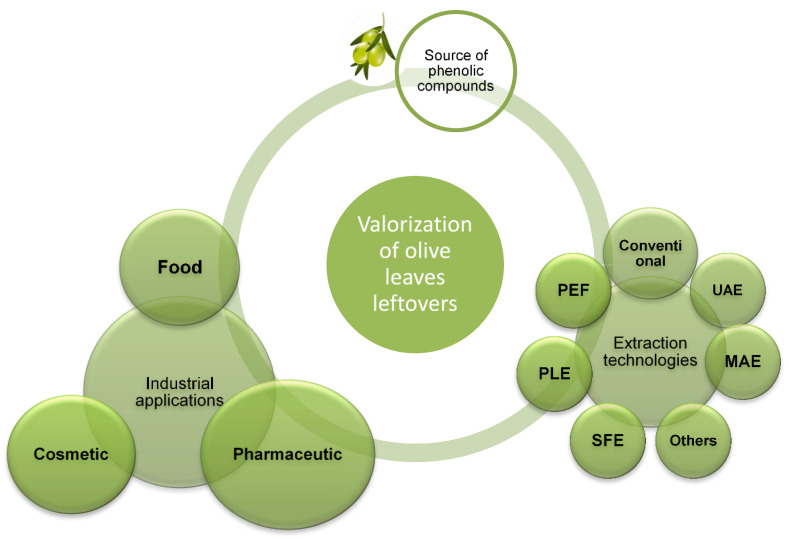
Valorization of olive leaves by a circular economy approach.

The accumulation and management of agro-industrial solid residues, such as olive leaves from pruning and oil processing systems, represents a serious problem from an economic and an environmental point of view. These residues are not only undesirable in terms of sustainability and environmental impact, but also create high costs for management and disposal. Conversely to the linear economy, in which agricultural by-products are mainly disposed of as combustion feedstock for biofuels, their valorization (that is the process of converting them into more useful products [131]) is nowadays regarded as central to the emerging bioeconomy (Figure 2). Indeed, these residual biomasses are rich in high-value compounds, which, as mentioned above, can be either used directly after extraction or exploited as ingredients with different applications in food, pharmaceuticals, and cosmetics, thus accelerating the implementation of the “Transforming our world: the 2030 Agenda for Sustainable Development” [132].

Nevertheless, the transition from a linear to a circular economy requires a cultural and structural change: a deep revision of and innovation in production, distribution, and environmentally sustainable consumption models [133]. For instance, regarding applications in the food sector, consumer acceptance of food enriched with olive leaves that are not part of the traditional diet must be carefully estimated. A recent case study for olive leaves has established that, despite the negative influence of food neophobia or technophobia, a core of sustainability-minded consumers interested in organic or local products would be favorable to the uptake of novel food made with upcycled ingredients in the market. This means that developing organic or local food products with olive leftovers can increase the probability of consumer acceptance because they are perceived as eco-compatible, as very close to cultural roots, and as a support for the local economy and local farmers [134,135]. Of course, suitable marketing policies would be of great importance in this sense, because indicating the benefits these foods could bring to health and the environment clearly in the label should help to deliver novel food to the greater public.

Continuous processes devoted to the recovery of phytochemicals and the production of energy from olive-oil industry waste and by-products must provide effective interaction between green technology and environmental and economic sustainability, which are consistent with the principles of the circular economy. Currently, operating plants exist in Spain and Italy that are able to produce up to 6000 kg of standardized polyphenol fractions and reutilize the residues of the process (water, olive cores, destoned pulp) into the same and/or other platforms according to the circular economy models [116]. However, more radical and systemic changes will be necessary for effective implementation of circular business models contributing to sustainable development in the Mediterranean olive sector, with adequate subsidies, common regulations, more collective actions for creating economies of scale, and marketing strategies to increase consumer awareness of bio-based products [136].

## 7. Conclusions

Olive leaves are the by-products of the agricultural and processing methods of olive crops. They are frequently unexplored, even though they are abundant in valuable bio-compounds (especially biophenols) and their disposal causes huge economic and environmental impacts. However, their bio-phenol profile and content is affected by agronomic factors (such as cultivar, leaf age, and degree of ripening) and climate conditions, showing a general trend of decreasing in the samples of trees cultivated in windy and humid air or in hot seasons and temperate regions. Care must be taken in harvesting the leaves as well as in selecting proper cultivars for obtaining specific types of biophenols.

The recovery of these valuable compounds, responsible for many health benefits, is a strategic challenge in valorizing an agro-industrial leftover in line with a circular economy approach; indeed olive leaf extracts have shown great market potential for industrial applications in the food, pharmaceutical, and cosmetic sectors. Of course, the selection of a viable and inexpensive technology to accommodate optimal extractions is a fundamental choice. Therefore, in the literature, numerous research studies have examined sustainable innovative technologies (i.e., MAE, UAE, etc.) to replace the conventional extraction systems in order to determine the best operative conditions (time, temperature, solvent to solid ratio, particle size etc.) for increasing the recovery of biophenols from olive leaves while saving time and cost.

## Figures and Tables

**Table 1:** Concentration levels of main phenolic compounds in olive leaves.

Class	Phenolic Compounds	Range (ng/mL Extract)	Ref	Range (mg/g dw)	Ref	Range (mg/g fw)	Ref
Secoiridoids	Oleuropein aglycone	45.7–548.1	[8]	0.09–0.27	[48]		
				0.011–0.13	[8]		
	Oleuropein glucoside	380–1548	[8]	0.72–1.48	[48]	1.29–2.11	[49]
				0.28–0.68	[50]		
				1.8–3.4	[49]		
				0.07–0.38	[8]		
	Demethyloleuropein	16.3–2121	[8]	0.18–0.40	[48]		
				0.0051–0.38	[8]		
	Oleuropein	50.4–23,485	[51]	15–81	[45]	30.46–71.12	[52]
		6750–126,940	[4]	43.4–122.3	[53]	0.081–0.753	[54]
		22,942	[55]	0.11–4.74	[56]	0.082–0.70	[57]
		19,880–73,650	[53]	21.5–106.5	[58]	40.3–99.3	[59]
		21,571–37,791	[8]	37.8–71.3	[48]	0.04–1.69	[60]
				0.65–14.08	[50]	69–108.6	[49]
				0.073–0.416	[61]	0.086–0.334	[61]
				7.8–13.5	[62]		
				0.79–13.65	[4]		
				169.32–210.65	[63]		
				0.60–0.78	[64]		
				0.65–1.41	[65]		
				14.1–40.4	[66]		
				0.50–22.42	[60]		
				3–48	[49]		
				11.84–14.01	[67]		
				67.05–74.81	[68]		
				65.57–69.91	[69]		
				63.35	[30]		
				4.68–10.08	[8]		
	Hydroxyoleuropein	380.2–886.9	[8]	0.30–1.95	[50]	0.02–0.06	[60]
				0.01–0.19	[60]		
				0.10–0.28	[8]		
	Ligstroside			0.78–0.99	[48]	1.18–4.16	[70]
				0.36–0.95	[50]	0.01–0.11	[60]
				0.01–0.80	[60]	1.35–2.13	[49]
				0.6–0.9	[49]		
	Methoxyoleuropein			0.051–0.186	[48]		
	Oleoside			0.031–0.051	[48]	0.05–0.08	[60]
				0.97–2.22	[50]		
				0.10–0.15	[60]		
	Secologanoside			0.92–1.49	[48]	0.28–0.37	[60]
				0.18–0.81	[60]		
	Verbascoside	914	[55]	0.62–2.23	[53]	0.47–1.26	[52]
		2.9–26	[8]	0.005–0.170	[56]	0.021–0.148	[54]
				2.91	[71]	0.36–2.31	[72]
				0.178–0.351	[62]	0.01–0.18	[60]
				1.98–4.40	[66]	1.5–2.7	[49]
				0.06–1.05	[60]		
				0.3–1.9	[49]		
				0.43–0.47	[68]		
				0.060–2.32	[8]		
	Isoverbascoside	91.6–3847	[8]	0.024–0.97	[8]		
	Elenolic acid glucoside			0.111–0.536	[48]		
				0.52–4.03	[50]		
Flavonoids	*Flavones*						
	Luteolin	71.5–2357	[51]	0.09–3.04	[45]	0.003–0.400	[54]
				0.0018–0.274	[56]	0.14–0.58	[60]
				0.006–0.025	[48]		
				0.220	[71]		
				0.14–0.63	[66]		
				0.05–1.18	[60]		
				0.07–0.60	[73]		
	Luteolin-7-*O*-glucoside	2370–9030	[4]	0.94–4.65	[53]	0.31–1.54	[60]
		3742	[55]	0.0083–0.819	[56]	8.9–10.6	[49]
		197.7–658.1	[8]	2.25	[71]		
				0.28–0.97	[4]		
				0.49–0.83	[64]		
				0.76–1.30	[65]		
				2.95–4.45	[66]		
				1.02–2.15	[60]		
				2.6–11.1	[49]		
				1.32–1.82	[69]		
				2.71	[30]		
				0.05–0.12	[8]		
	Luteolin glucoside			1.53–2.62	[48]	0.18–0.73	[60]
				0.60–1.54	[50]		
				0.32–0.90	[60]		
	Luteolin diglucoside	12.4–104.8	[8]	0.15–0.25	[48]	0.01–0.03	[60]
				0.02–0.05	[60]		
				0.003–0.03	[8]		
	Luteolin rutinoside			0.19–0.41	[48]	0.02–0.11	[60]
				0.14–0.22	[60]	0.91–1.14	[49]
				0.48–0.99	[49]		
	Luteolin-4′-*O*-glucoside			0.234–0.493	[62]		
				0.22–0.30	[64]		
				0.25–0.31	[65]		
				19.16–31.02	[73]		
	Apigenin	31.9–198	[51]	0.093	[71]		
				0.023–0.094	[66]		
				0.038	[73]		
	Apigenin-7-*O*-glucoside	2147	[55]	0.73–3.01	[53]	0.013–1.46	[57]
				0.0094 –2.476	[56]		
				0.347	[71]		
				0.28–0.85	[66]		
				1.00–2.06	[73]		
	Apigenin diglucoside	11.2–31.6	[8]	0.09–0.26	[49]	0.12	[49]
				0.0028–0.0081	[8]		
	Apigenin rutinoside	4.9–81.1	[8]	0.033–0.061	[48]	0.10–0.13	[60]
				0.14–0.31	[64]	0.74	[49]
				0.25–0.39	[65]		
				0.06–0.17	[60]		
				0.33–0.66	[49]		
				0.0012–0.013	[8]		
	Diosmetin	14.5–159	[51]				
	Chrysoeriol-7-*O*-glucoside			0.058–0.173	[48]		
	Flavanones						
	Eriodictyol			0.0068	[71]		
	Hesperidin			0.304	[71]		
	Naringenin					0.029–4.05	[57]
	Neohesperidin			0.39–0.99	[49]	0.78–1.13	[49]
	*Flavonols*						
	Rutin	357	[55]	0.02–1.65	[45]	0.034–1.331	[54]
				0.0026–0.825	[56]	0.03–0.14	[60]
				0.18–0.29	[48]		
				0.35–0.98	[66]		
				0.19–0.39	[60]		
				2.01–2.94	[68]		
				0.43–0.85	[73]		
	Quercetin rutinoside	14.89	[55]	0.078–0.245	[64]		
		191–1379	[8]	0.16–0.25	[65]		
				0.047–0.37	[8]		
	Quercetin galactoside	30.57	[55]				
	Quercetin glucoside	0–115.3	[8]	0–0.003	[8]		
	Quercetin rhamnoside	0–86.9	[8]	0–0.0023	[8]		
	Quercetin	0.021–247	[51]	0.02–0.37	[45]	0.002–2.73	[57]
				0.18–0.76	[53]		
				0.040	[71]		
				0.0008–0.0021	[73]		
	Hyperoside			0.067	[71]		
	Kaempferol			0.0029	[71]	0.039–8.86	[57]
	Isorhamnetin					0.052–2.41	[57]
	*Flavanonols*						
	Taxifolin			0.0082	[71]		
	*Flavan-3-ols*						
	Catechin			0.18–0.36	[66]		
				0–0.0376	[56]	0.16–3.29	[57]
	Epicatechin	21.7	[55]				
Simple	*Phenylethanoids*						
phenols	Tyrosol	1681	[55]	0.2–1.95	[45]	0.001–0.036	[54]
				0.0021–0.175	[56]		
				0.033–0.088	[66]		
	Tyrosol glucoside			0.76–1.44	[48]		
	Hydroxytyrosol	9.2–3682	[51]	0.02–27.20	[45]	0.189–1.313	[54]
		10,925	[55]	0.0054 –0.290	[56]		
				0.56–2.94	[58]		
				0.21–0.60	[66]		
				0.33–0.40	[67]		
				0.87–2.19	[68]		
	Hydroxytyrosol glucoside	570–2357	[8]	1.61–2.37	[48]	1.01–4.60	[52]
				0.57–1.41	[60]	0.85–3.41	[72]
				0.13–0.58	[8]	0.82–1.03	[60]
	*Hydroxycinnamic acids*						
	Caffeic acid	201.5	[55]	0.34–2.11	[45]	0.014–0.85	[57]
				0.0026–0.432	[56]	0.04–0.10	[72]
				0.015	[71]		
				0.27–0.39	[68]		
	*p*-Coumaric acid	0.81	[55]	0.15–2.34	[45]	0.003–0.49	[57]
				0.081	[71]		
	Chlorogenic acid	30.72	[55]	0.0027	[71]		
				0.39–0.47	[68]		
	Cinnamic acid					0.005–1.09	[57]
	Ferulic acid			0.003–0.25	[45]	0.016–0.79	[57]
				0.046	[71]		
	Syringic acid			0.002–0.09	[45]	0.017–1.16	[57]
				0.0028	[71]		
	*hydroxybenzoic acids*						
	Vanillic acid			0.0032–0.257	[56]		
				0.016	[71]		
	Vanillin	0.47	[55]	0.0016–0.147	[56]		
	Sinapic acid			0.0049	[71]		
	Gallic acid	1.73	[55]	1.15–3.04	[45]	0.68–1.45	[57]
				0.0026	[71]		
	Protocatechuic acid			0.40–3.23	[45]		
				17.48	[71]		
	2,5-Dihydroxybenzoic acid			0.0055	[71]		
	3,4-Dihydroxybenzoic acid					0.11–2.07	[57]
	4-Hydroxybenzoic acid			0.015	[71]		
	3,4-Dihydroxyphenylacetic acid			0.47	[71]		
Lignans	Pinoresinol			0.004	[71]		

Dw: dry weight; fw: fresh weight.

**Table 2:** Principal innovative extraction technologies applied to olive leaves: principle and operating parameters.

Extraction Technique	Principle/Mechanism	Operating Parameters	Ref
Ultrasound Assisted Extraction (UAE)	Acoustic cavitation, acoustic pressure in addition to hydrostatic pressure, micro-jetting and micro-streaming effects, cell wall breakdown and particle dislodgment	US power and frequency, amplitude, temperature, time, solvent, liquid-solid ratio	[62,73,101,102]
Microwave Assisted Extraction (MAE)	Ionic conduction and dipole rotation, conversion of electromagnetic energy to thermal energy, cell wall disruption by high pressure	MW power, time, temperature, solvent, liquid-solid ratio	[86,103,104]
Supercritical Fluid Extraction (SFE)	Increased density and reduced viscosity of extraction fluid at temperature and pressure above critical point, increased penetration and mass transfer	Supercritical fluid (CO_2_, the most commonly used), co-solvent, temperature, pressure, fluid flow rate	[105,106,107]
Pressurized Liquid Extraction (PLE)	Increased solubility and diffusion rate at elevated temperature (above boiling point) under pressurized condition, reduction of solvent viscosity, increased mass transfer	Number and duration of extraction cycles, solvent, pressure, temperature liquid-solid ratio	[23,53,108]
Pulsed Electric Field (PEF) extraction	Electro-permeabilization by electromechanical force, electroporation of cell membrane, increased mass transfer	Electric field intensity/input energy, pulse duration (μs) and number, extraction chamber geometry	[64,65]

US: ultrasound. MW: microwave.

**Table 3:** Recent patent application (2011–2021) of olive leaf extracts in food, pharmaceutic, and cosmetic sectors.

Number	Date	Patent Target	Sector
KR101027385B1	2011	Method for breeding livestock using olive leaves in order to produce low-fat, low-cholesterol, and high-unsaturated-fatty-acids meat.	Food industry
HRP20090650A2	2011	Preparation method of a formulation based on resveratrol and extracts from pomegranate, olive leaf, and cinnamon used as an effective therapy for oxidative stress.	Pharmaceutic industry
US2013272974A1	2012	Preparation and purification of a granule powder from olive leaves containing a minimum of 25% hydroxytyrosol.	Food industry
EP2462991A1	2012	Use of olive leaf products for the treatment of bacterial and/or fungal nail infections.	Pharmaceutic/cosmetic industry
CN103110549A	2013	Method for preparing an olive leaf extract microemulsion rich in hydroxytyrosol used for preventing fat and oil from rotting.	Food industry
CN103798504A	2014	Method for preparing biological feed by solid-fermenting olive leaves with a high nutritional value.	Food industry
CN104127469A	2014	Application of the olive leaf crude extract in the preparation of a drug or a health-care product, which is used for treating or preventing the inhibition of immunologic function or inhibition of medullary hematopoiesis caused by drugs in radiotherapy and/or chemotherapy.	Pharmaceutic industry
CN105899221A	2014	Preparation process of a dermatological composition comprising compounds of algae (e.g., Arthrospira platensis) and olive leaf for preventing dermatological microbial infections.	Pharmaceutic/cosmetic industry
CN104926615A	2015	Enzymolysis process for the crude extraction of olive leaf oleuropein to prepare hydroxytyrosol.	Pharmaceutic/food industry
CN105011162A	2015	Environmentally friendly and zero-pollution technology for the production of olive leaf essence which is small in nutrient loss and high in content and yield.	Food industry
CN104622934A	2015	Application of olive leaf crude extract in the preparation of drugs for treating sphagitis.	Pharmaceutic industry
KR20150115186A	2015	Application of olive leaves in preparation or screening of therapeutic drugs for treating acute or chronic cough.	Pharmaceutic industry
CN104971082A	2015	Application of olive leaves in preparation or screening of therapeutic drugs for treating ozostomia.	Pharmaceutic industry
CN104768561A	2015	Use of oleuropein and hydroxytyrosol extracted from olive leaves in a variety of applications to prevent, reduce symptoms of, and treat conditions related to insulin sensitivity, including type 2 diabetes.	Pharmaceutic industry
US2016106128A1	2016	Method of producing high quality olive leaf powders, which can be included in various food, pharmaceutical, cosmetic, and antimicrobial compositions, using infrared dry blanching, drying, and milling.	Pharmaceutic/food industry
CN105997703A	2016	The invention provides an olive leaf extract which comprises any one or a combination of 5–50% by mass. The invention further provides a preparation method of an olive leaf extract rich in hydroxytyrosol, oleuropein, and verbascoside with high skin anti-aging effect.	Pharmaceutic/cosmetic industry
CN106421741A	2017	Preparation method of a water-soluble olive leaf extract health-care product for regulating blood glucose and insulin sensibility.	Pharmaceutic industry
CN108041522A	2017	Preparation method of olive leaf tea powder jelly based on microwave assisted enzymolysis.	Food industry
CN107753566A	2017	Method for preparing olive leaf medicated liquid	Pharmaceutic industry
CN107412077A	2017	Method for preparing an ultraviolet stress deep-fermentation olive leaf extract for blocking ultraviolet rays and repairing skin injury.	Pharmaceutic/cosmetic industry
CN107019672A	2017	Preparation method of long-circulation lipidosome of an olive polyphenol extract rich in hydroxytyrosol and verbascoside.	Pharmaceutic industry
CN106619324A	2018	Preparation method of an anti-allergic mask containing olive leaf extract which can relieve the neurosensory activity of a sensitive skin symptom and improve the immunity of the skin, having double anti-allergic effects.	Pharmaceutic/cosmetic industry
CN109370866A	2019	Method for preparing vinegar from olive juice and olive leaves rich in nutrients and with health-care functions.	Food industry
CN110463803A	2019	Method for preparing de-bittered olive leaf functional fermented tea with white ginseng fungus, which enriches and enhances the types and contents of functional components.	Food industry
CN109364090A	2019	Application of olive leaf extract to prepare drugs for preventing and treating fetal alcohol syndrome.	Pharmaceutic industry
US2020054053A1	2020	Method for preparing olive leaf powder based on vacuum freeze-drying to effectively preserve active substances.	Food industry
AU2020100302A4	2020	Process to extract olive leaf antioxidants together with anti-viral, anti-fungal and anti-bacterial compounds, to manufacture either an olive leaf tincture, hand-wash gel, no-rinse hand sanitiser, and/or a hand soap.	Pharmaceutic/cosmetic industry
CN110999908A	2020	The invention discloses application of hydroxytyrosol acetate from olive leaves to prepare an effective bactericide, usable as a potential antibacterial agent.	Pharmaceutic industry
US10702550B1	2020	Use of olive leaf extract to synthesize silver nanoparticles (AgNPs).	Pharmaceutic industry
CN112107607A	2021	Application of olive leaf extract in preparing a medicine for treating avian influenza.	Pharmaceutic industry
CN112076242A	2020	Use of olive leaf extract in preparing therapeutic drugs for treating swine fever or African swine fever.	Pharmaceutic industry
US10925916B2	2021	Invention of films made with edible polymers and containing a portion of finely ground olive leaf powder (OLP) suitable for preparing a hot or cold beverage and delivering an unexpectedly powerful quantity of natural anti-oxidants in the form of polyphenols.	Food industry
WO2021053259A1	2021	Preparation method of a functional food composition with an oleuropein content of 80–85% by weight from olive leaves to use in the food, cosmetic or pharmaceutical industries.	Pharmaceutic/Food industry
